# Cellular responses of *Saccharomyces cerevisiae* at near-zero growth rates: transcriptome analysis of anaerobic retentostat cultures

**DOI:** 10.1111/j.1567-1364.2011.00750.x

**Published:** 2011-09-26

**Authors:** Léonie GM Boender, Antonius JA Maris, Erik AF Hulster, Marinka JH Almering, Ida J Klei, Marten Veenhuis, Johannes H Winde, Jack T Pronk, Pascale Daran-Lapujade

**Affiliations:** 1 Kluyver Centre for Genomics of Industrial FermentationDelft, The Netherlands; 2 Department of Biotechnology, Delft University of Technology, DelftThe Netherlands; 3 Molecular Cell Biology Group, Groningen Biomolecular Sciences and Biotechnology Institute, University of GroningenHaren, The Netherlands

**Keywords:** retentostat, *Saccharomyces cerevisiae*, near-zero growth rates, quiescence, chronological ageing

## Abstract

Extremely low specific growth rates (below 0.01 h^−1^) represent a largely unexplored area of microbial physiology. In this study, anaerobic, glucose-limited retentostats were used to analyse physiological and genome-wide transcriptional responses of *Saccharomyces cerevisiae* to cultivation at near-zero specific growth rates. While quiescence is typically investigated as a result of carbon starvation, cells in retentostat are fed by small, but continuous carbon and energy supply. Yeast cells cultivated near-zero specific growth rates, while metabolically active, exhibited characteristics previously associated with quiescence, including accumulation of storage polymers and an increased expression of genes involved in exit from the cell cycle into G_0_. Unexpectedly, analysis of transcriptome data from retentostat and chemostat cultures showed, as specific growth rate was decreased, that quiescence-related transcriptional responses were already set in at specific growth rates above 0.025 h^−1^. These observations stress the need for systematic dissection of physiological responses to slow growth, quiescence, ageing and starvation and indicate that controlled cultivation systems such as retentostats can contribute to this goal. Furthermore, cells in retentostat do not (or hardly) divide while remaining metabolically active, which emulates the physiological status of metazoan post-mitotic cells. We propose retentostat as a powerful cultivation tool to investigate chronological ageing-related processes.

## Introduction

Microbial growth in natural environments is generally limited by nutrient availability. Indeed, 80% of microbial life on Earth has been estimated to exist as slowly or non-proliferating cells (Brock, 1971; Lewis & Gattie, 1991). Insight into the physiology of (near-)zero growth rate is therefore very relevant for understanding microbial life in natural environments. Furthermore, in industrial biotechnology it may contribute to uncoupling microbial product formation from growth, thereby preventing formation of excess biomass. However, research on microbial physiology and cellular regulation has predominantly focused on cells that display discernable growth, and even in extensively studied organisms such as the yeast *Saccharomyces cerevisiae*, extremely slow growth has not been exhaustively studied. Constraints in the experimental accessibility of near-zero growth rates form a key factor in this lack of information (Pirt, 1987).

In conventional glucose-grown batch cultures (e.g. shake flask cultures) of *S. cerevisiae*, a predominantly fermentative growth phase on glucose is followed by a respiratory growth phase in which products of glucose fermentation (predominantly ethanol) are metabolized. When these are depleted, the culture moves into stationary phase. During transition to stationary phase, *S. cerevisiae* executes a complex reprogramming of its cellular biology that involves down-regulation of protein synthesis, increased stress tolerance, thickening of the cell wall and accumulation of storage polymers such as glycogen, trehalose and triacylglycerol (for reviews see Werner-Washburne *et al*., 1993; Herman, 2002; Gray *et al*., 2004; Smets *et al*., 2010). This robust phenotype is commonly referred to as quiescence (Werner-Washburne *et al*., 1996; Gray *et al*., 2004). Quiescent cells survive by the slow mobilization of storage compounds whose depletion ultimately leads to deterioration and cell death.

In batch cultures, the continuous and usually fast progression from exponential growth to stationary phase makes it difficult to specifically study the physiology of near-zero growth rates. In contrast to batch cultures, chemostat cultures enable steady-state nutrient-limited growth at submaximal specific growth rates (Monod, 1950; Novick & Szilard, 1950; Herbert *et al*., 1956). Chemostat cultivation of *S. cerevisiae* has been extensively used for genome-wide studies on the impact of specific growth rate on physiology and for genome-wide expression studies (Regenberg *et al*., 2006; Castrillo *et al*., 2007; Brauer *et al*., 2008; Daran-Lapujade *et al*., 2009a). These studies revealed that specific growth rate strongly influences expression of a large number of yeast genes. For example, the previously identified STRE (general stress response) regulon was in fact consistently upregulated at low specific growth rates, even in ‘non-stressed’ cultures (Regenberg *et al*., 2006; Castrillo *et al*., 2007; Brauer *et al*., 2008). However, in these chemostat studies, the lowest specific growth rates investigated were between 0.02 and 0.07 h^−1^. The long time period required to reach steady state and the occurrence of ‘feast-famine’ dynamics due to dropwise feeding of medium present important practical constraints for studies at lower specific growth rates (Herbert *et al*., 1956; Daran-Lapujade *et al*., 2009a). As a consequence, chemostat cultivation does not provide experimental access to the physiology of extremely slow growth.

The importance of studying non-growing, but metabolically active cells extends beyond microbial physiology, as their physiological status resembles that of post-mitotic cells in multicellular eukaryotes. Non-proliferative cells such as nerve cells and heart muscle cells have exited the replicative cell cycle into a G_0_ resting phase, but remain metabolically active for long periods of time. Since the isolation of mutants that are unable to exit G_0_ and the identification of the Ego complex responsible for this process (Dubouloz *et al*., 2005), existence of a G_0_ phase in quiescent yeast cells is now commonly accepted. Due to its unprecedented experimental accessibility, *S. cerevisiae* has become a popular eukaryotic laboratory model for studying ageing in post-mitotic cells. However, the question has been raised whether the most commonly applied cultivation method in ageing studies, which consists of starving yeast cells in stationary phase, provides an adequate model for survival and longevity of metabolically active post-mitotic cells (Gershon & Gershon, 2000a, b).

Retentostat cultures were developed with the specific aim to study microbial physiology at extremely low specific growth rates (Herbert, 1961; van Verseveld *et al*., 1986). In essence, retentostats are chemostats in which complete biomass retention in the culture is accomplished by removing the effluent through a filter unit or using an external biomass-recycling device. When grown at a fixed dilution rate on a medium in which the energy substrate is growth limiting, biomass accumulation will lead to a progressive decrease of the biomass-specific consumption rate of the energy substrate. As a result, the substrate consumption rate will asymptotically approach the substrate requirement for maintenance processes (e.g. turnover of damaged cellular components, maintenance of chemiosmotic potentials). Consequently, growth will cease while starvation is prevented because substrate continues to be fed. Although retentostats have been used to study the basic physiology of microorganisms at near-zero specific growth rates (Chesbro *et al*., 1979; van Verseveld *et al*., 1986; Tappe *et al*., 1996), they have not yet been applied in combination with genome-wide analysis tools.

We recently implemented retentostat cultivation for growth of *S. cerevisiae* under anaerobic, glucose-limited conditions and demonstrated that specific growth rates below 0.005 h^−1^, corresponding to a doubling time of 139 h, could be reproducibly reached and studied while the large majority of the cells remained viable (Boender *et al*., 2009). The goal of the present study was to investigate genome-wide transcriptional responses of *S. cerevisiae* at extremely low specific growth rates and to compare it with available data on gene expression in faster-growing and stationary-phase yeast cultures. To this end, we analysed the transcriptome of retentostat cultures during the progressive decrease of specific growth rate. The results were then combined with chemostat-based data to enable the dissection of transcriptional responses to extremely low specific growth rate from general growth rate-dependent expression. Interpretation of the transcriptome data was verified by analyses of cellular composition and structure.

## Materials and methods

### Strain, media and cultivation conditions

The prototrophic laboratory strain *S. cerevisiae* CEN.PK113-7D (*MAT*a *MAL*2-8^c^ *SUC*2, obtained from Dr P. Kötter, Frankfurt, Germany) was used in the present study. Stock cultures were prepared from a shake flask culture in YPD (Yeast Peptone Dextrose) medium (Sherman, 1991) grown to stationary phase, by addition of glycerol (20% v/v) and storage of 2 mL aliquots in sterile vials at −80 °C. Pre-cultures for retentostat cultivation were made by inoculating a frozen stock culture in 500 mL shake flasks with 100 mL synthetic medium (Verduyn *et al*., 1992) at pH 6 with 2% glucose.

The same synthetic medium containing 5% glucose, complemented with the anaerobic growth factors ergosterol (final concentration, 10 mg L^−1^) and Tween-80 (final concentration, 420 mg L^−1^), and with the antifoaming agent Struktol J673 (final concentration 0.03% w/w; Schill and Seilacher AG, Hamburg, Germany, sterilized separately at 120 °C), was used for the retentostat cultures (Verduyn *et al*., 1990a). To keep medium composition constant during long-term cultivation, 40 L batches of medium were prepared, filter-sterilized and used for single retentostat experiments. Vitamins and anaerobic growth factors were added to the medium reservoirs as described previously (Verduyn *et al*., 1990b).

Anaerobic glucose-limited retentostats with a dilution rate of 0.025 h^−1^ and a working volume of 1.4 L were operated as described previously (Boender *et al*., 2009). Duplicate retentostat cultures were started from independent anaerobic, glucose-limited chemostat cultures grown at the same dilution rate. Retentostat cultivation was started when macroscopic measurements (culture dry weight and specific carbon-dioxide production rate) changed by less than 2% during two consecutive volume changes, by redirecting the effluent through an AppliSense Sample Filter assembly (0.22 μm pore size; Applikon, Schiedam, the Netherlands) rather than through the standard effluent tube. Fermenters were equipped with Norprene tubing and O-rings to avoid oxygen diffusion, and both the fermenter and medium vessel were continuously sparged with ultra-pure nitrogen gas containing <5 p.p.m. oxygen (Linde, Schiedam, The Netherlands), as described previously (Visser *et al*., 1990). Culture purity was routinely checked by phase-contrast microscopy and by plating on glucose-containing synthetic medium with 20 mM LiCl (Daran-Lapujade *et al*., 2009b).

### Analytical methods

Gas analysis, biomass dry weight measurements and metabolite analysis were performed as described previously (Boender *et al*., 2009). For analysis of trehalose and glycogen, exactly 20 mL of culture broth was centrifuged (4 °C, 5 min at 10 000 ***g***), washed once with ice-cold demineralized water and resuspended in demineralized water to an exact concentration of 5.0 g L^−1^ biomass. After storage of these samples at −20 °C, trehalose and glycogen assays (Parrou & Francois, 1997) were performed. Glucose released by glycogen and trehalose conversion was determined using the UV method based on Enzyplus™ kit EZS781 (BioControl, Southampton, UK). Trehalose and glycogen were determined in triplicate for each sample.

### Assessment of culture viability

Five-millilitre culture samples were diluted with 20 mL of 10 mM Na-Hepes buffer (pH 7.2) with 2% glucose. The total cell concentration was measured with a Coulter counter using a 50 μm orifice (Multisizer II; Beckman, Fullerton, CA). Viability was assessed using the LIVEDEAD® Yeast viability kit (Invitrogen, Carlsbad, CA) following the supplier's instructions. After centrifugation (16 000 ***g***, 5 min), resuspension in incubation buffer (10 mM Na-Hepes buffer pH 7.2, 2% glucose) and addition of 1 μl of Fun1® dye (10 mM in DMSO), the cell suspension was incubated for 1 h at 30 °C. Metabolically active cells were identified and counted based on the formation of red cylindrical intravacuolar structures as observed using a fluorescence microscope (Imager-D1; Carl-Zeiss, Oberkochen, Germany) equipped with Filter Set 09 (FITC LP Ex. BP 450-490 Beamsp. FT 510 Em. LP 515; Carl-Zeiss). At least 200 cells were counted and used to calculate viability. Standard deviation of viability assays was typically below 10%.

### Flux calculations

The accumulation of biomass during retentostat cultivation under ‘ideal’ conditions (growth-rate independent maintenance-energy requirements, no lysis or loss of viability) is described by Eqn. (1) (van Verseveld *et al*., 1986; Boender *et al*., 2009), in which *C*_*x*_ denotes the biomass concentration in (g L^−1^), *D* is the dilution rate (h^−1^), *C*_*s*,in_ is the glucose concentration in the medium vessel (g L^−1^) and *C*_*s*_ is the residual glucose concentration (g L^−1^). The maintenance coefficient *m*_*s*_ used in these calculations was 0.50 mmol glucose g^−1^ h^−1^ and the maximum biomass yield on glucose $Y_{\mathrm{sx}}^{\max}$ was 0.097 g g^−1^ (Boender *et al*., 2009).


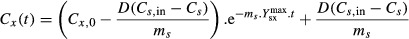


To calculate specific growth rate in the retentostat cultures, the measured total biomass concentrations (which included both viable and non-viable cells) were fitted with *C*_*x*_ = A.e^*B*.*t*^ + C, which is of the same shape as Eqn. (1) (matlab; The MathWorks, Natick, MA; function fminsearch for minimizing sum of squares of errors by varying A, B and C). Having fitted the coefficients A, B and C, the derivate (d*C*_*x*_/d*t*) could be calculated. As only viable cells can replicate and by assuming that no lysis (i.e. loss of measurable biomass) occurs, the specific growth rate was calculated from Eqn. (2).


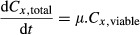


### Electron microscopy

At *t* = 0, 7, 14 and 22 days of retentostat cultivation, samples were taken for transmission electron microscopy. One millilitre of culture was centrifuged (room temperature, 5 min at 16 000 ***g***), washed twice with sterile water and fixed with either 1.5% (w/v) potassium permanganate (KMnO_4_) or cold 3% (v/v) electron microscopy-grade glutaraldehyde in 0.1 M Na-cacodylate buffer pH 7.2. The KMnO_4_ fixation was incubated for 20 min at room temperature, centrifuged (room temperature, 5 min at 16 000 ***g***) and washed repeatedly with sterile water until the supernatant was colourless. The glutaraldehyde fixation was incubated on ice for 2 h. Afterwards, the sample was centrifuged (room temperature, 5 min at 16 000 ***g***) and washed once with cacodylate buffer. Both fixations were stored at 4 °C until the cells were processed. To study cellular morphology, KMnO_4_-fixed cells were embedded in Epon 812 (Shell, The Hague, the Netherlands) and examined in an electron microscope (CM12; Philips) (Waterham *et al*., 1994). To visualize glycogen, phosphotungstic-acid staining was performed on the glutaraldehyde-fixed cells (Farragiana & Marinozzi, 1979). Reagents were obtained from Sigma-Aldrich Chemie (Zwijndrecht, the Netherlands).

### Microarrays and transcriptome analysis

Independent duplicate retentostat cultures were subjected to microarray analysis at four time points after switching the effluent line to the filter unit (2, 9, 16 and 22 days). Microarray analysis of independent, triplicate anaerobic glucose-limited chemostat cultures grown at a specific growth rate of 0.025 h^−1^ (*t* = 0) were also performed as part of this study, resulting in a dataset of 11 arrays. These array data can be retrieved from Genome Expression Omnibus (GEO, http://www.ncbi.nlm.nih.gov/geo/) with series number GSE22574. These data were combined with previously published microarray datasets obtained from chemostat cultures grown under identical conditions, but at specific growth rates of 0.03, 0.05, 0.1 and 0.2 h^−1^ (Fazio *et al*., 2008; Knijnenburg *et al*., 2009). The only notable difference was the glucose concentration in the feed of 25 g L^−1^ for chemostat cultures at 0.03, 0.05, 0.1 and 0.2 h^−1^ and of 50 g L^−1^ for chemostat cultures at 0.025 h^−1^ and retentostat cultures. For specific growth rates of 0.03, 0.1 and 0.2 h^−1^, microarray data were derived from three independent replicates, whereas cultures at 0.05 h^−1^ were performed in duplicate, thereby resulting in a final dataset of 22 microarrays. These additional chemostat-based microarray data are available from ArrayExpress with the accession number E-MTAB-78 (http://www.ebi.ac.uk/microarray-as/ae/) and from GEO with the accession number GSE11452. Sampling and quenching of biomass, RNA isolation, probe preparation and hybridization to Affymetrix GeneChip® microarrays (Santa Clara, CA) were performed as described previously (de Nicola *et al*., 2007). Data acquisition, quantification of array images and data filtering were performed with the affymetrix genechip® Operating Software version 1.2. Before comparison, all arrays were globally scaled to a target value of 300 using the average signal from all gene features.

To eliminate insignificant variations, genes with expression values below 12 were set to 12 and genes for which maximum expression was below 20 in all 22 arrays were discarded. From the 9335 transcript features on the YG-S98 arrays, a filter was applied to extract 6383 yeast open reading frames (Boer *et al*., 2003). For additional statistical analyses, Microsoft Excel running the edge (version 1.1.208) add-in was used (Storey *et al*., 2005) for a time-course differential expression analysis. To determine the genes called significantly changed according to edge, a *P*-value of 0.01 was used (*q*-value 0.000188). *K*-means clustering of the genes with significantly changed expression levels was subsequently performed using genedata expressionist pro® version 3.1 (Genedata, Basel, Switzerland). The *k*-means algorithm used positive correlation as distance metric. The maximum number of iterations was set to 1000. Each cluster was consulted for enrichment in functional annotation and significant transcription factor (TF) binding [experimentally identified by Harbison *et al*. (2004) as described previously (Knijnenburg *et al*., 2007)]. In addition, specific TF binding sites (BS) absent from the Harbison dataset were analysed using web-based Regulatory Sequence Analysis Tools (Van Helden *et al*., 2000). The enrichment factor (EF) for BS within promoter regions of specific groups of genes was computed as follows:

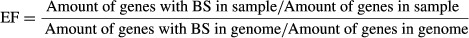


A set of glucose-responsive genes (Kresnowati *et al*., 2006) used as a reference is accessible from GEO with the accession number GSE3821. Statistical significance of the over-representation of these genes in subsets of yeast genes was computed as described in (Knijnenburg *et al*., 2007), replacing functional categories by the reference set of glucose-repressible genes.

## Results

### Cultivation at near-zero specific growth rates in retentostats

**Fig 1:**
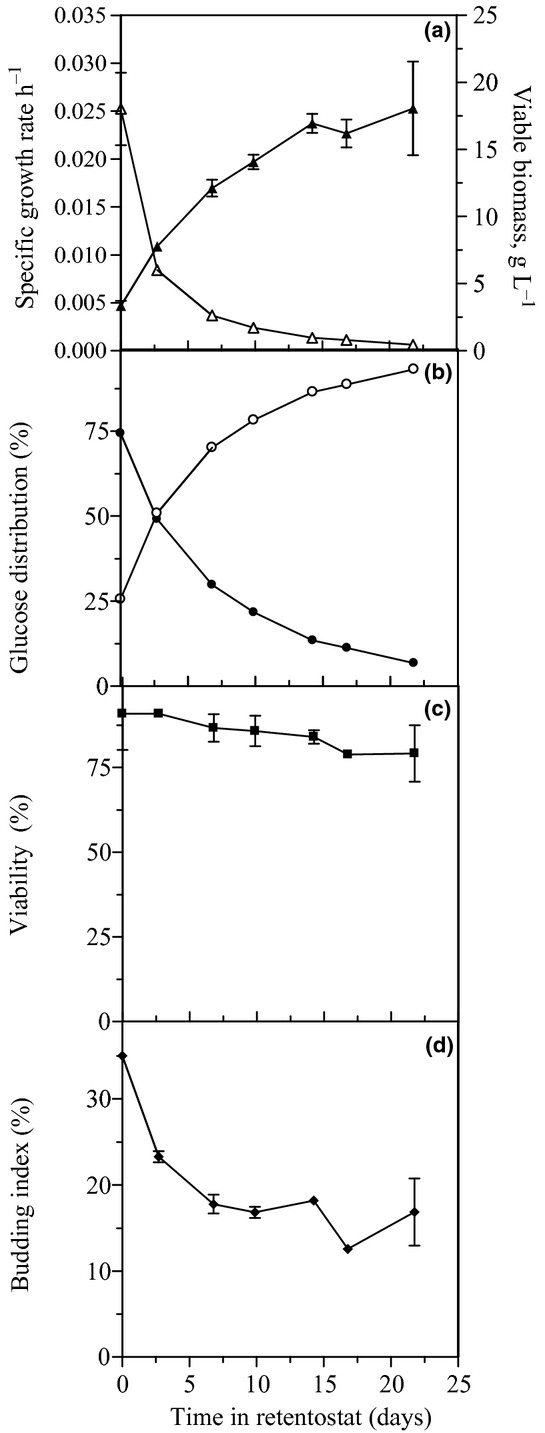
Physiology of anaerobic retentostat cultures. A steady-state anaerobic chemostat culture (dilution rate, 0.025 h^−1^) was switched to retentostat mode at *t* = 0. Data points represent average ± mean deviation of measurements on two independent cultures. (a) The specific growth rate during retentostat cultivation (△) and viable biomass concentration (estimated from fluorescent staining) (▲). (b) Distribution of glucose (%) between cellular maintenance (*m*_*s*_·*C*_*x*,viable_, ○) and growth (μ·*C*_*x*,viable_/$Y_{\mathrm{sx}}^{\max}$, ●). (c) Viability estimated from fluorescent staining. (d) Budding index.

Physiology and gene expression of *S. cerevisiae* CEN.PK113-7D at near-zero specific growth rates were studied in anaerobic, glucose-limited retentostat cultures (Boender *et al*., 2009). When the effluent flow of anaerobic, glucose-limited chemostat cultures (dilution rate, 0.025 h^−1^) was redirected through a filter probe (Boender *et al*., 2009), biomass accumulated in the cultures (Fig. 1a), thereby increasing the fraction of glucose that was used to meet maintenance-energy requirements (*m*_*s*_·*C*_*x*,viable_) and decreasing glucose availablity for growth (Fig. 1b). Prolonged growth in retentostats led to a partial loss of viability (Fig. 1c, Boender *et al*., 2009). Although viability was estimated as metabolic activity using a fluorescent stain, colony forming units assays revealed a similar decrease in viability (data not shown). When viability was used to calculate viable biomass concentrations in the retentostats, these closely fitted model-based predictions [Materials and methods, Eqn. (1)]. Over 22 days of retentostat cultivation, the estimated specific growth rate progressively decreased to 0.0006 ± 0.0001 h^−1^ (Fig. 1a) and the budding index decreased to 15% (Fig. 1d), which is a typical value for non-growing *S. cerevisiae* (Lewis *et al*., 1993).

### Transcriptome analysis: data quality and datasets integration

**Fig 2:**
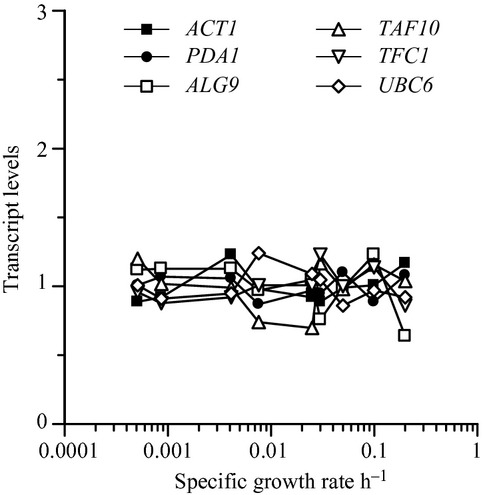
Mean-normalized expression levels of house-keeping genes during anaerobic retentostat culture and chemostat culture at various growth rates.

Transcriptome analysis was performed on independent duplicate retentostat cultures at selected time points. Average deviation of the mean of transcript data from replicate retentostats was around 14%, which is similar to the reproducibility usually observed in replicate analyses of steady-state chemostat cultures (Daran-Lapujade *et al*., 2004). Transcript levels were calculated by normalizing fluorescence outputs corresponding to individual genes on each microarray to its overall fluorescence (normalization method also known as global scaling). This method may impede accurate estimation of changes in expression when the mRNA pool undergoes massive changes, for example, during transfer from exponential to stationary phase in batch cultures (Van de Peppel *et al*., 2003). However, transcript levels of widely used house-keeping genes (*ACT1* and *PDA1*) and other genes whose expression has recently been shown to be steady throughout a variety of cultivation conditions (*ALG9*, *TAF10*, *TFC1* and *UBC6*, Teste *et al*., 2009) remained remarkably constant during the retentostat runs (coefficient of variation below 20%, Fig. 2). These results indicate that retentostat cultivation did not cause changes in the mRNA pools that precluded use of the standard normalization protocol. Samples were taken in chemostat before starting the retentostat culture (*t* = 0 days), and 2, 9, 16 and 22 days after switching to retentostat (corresponding respectively to growth rates of approximately 0.0084 h^−1^, 0.0024 h^−1^, 0.0011 h^−1^ and 0.00063 h^−1^).

**Fig 3:**
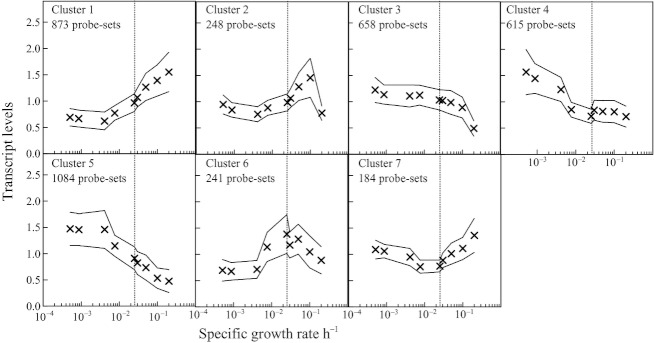
*K*-mean clustering of the genes with significantly changed expression as a function of growth rate (*q*-value below 0.000188, see Materials and methods). Transcript data for growth rates above 0.025 h^−1^ (indicated by the dashed line) were obtained from Fazio *et al*. (2008) and Cipollina *et al*. (2008).

Chemostat-based transcriptome studies on *S. cerevisiae* (Regenberg *et al*., 2006; Castrillo *et al*., 2007; Fazio *et al*., 2008) have identified many genes whose expression is tightly correlated with specific growth rate, such as genes involved in protein synthesis and nucleotide metabolism (Regenberg *et al*., 2006; Castrillo *et al*., 2007; Fazio *et al*., 2008). To investigate gene expression over a broader range of specific growth rates, we combined our retentostat-based transcriptome data with transcriptome data from chemostat cultures grown at specific growth rates ranging from 0.03 to 0.20 h^−1^ (Cipollina *et al*., 2005; Fazio *et al*., 2008). Except for the range of specific growth rates and the glucose concentration in the feed (see Materials and methods section), culture parameters (yeast strain, anaerobicity, nutrient limitation and growth medium, temperature, pH, mRNA sampling and microarray protocols) in the chemostat studies were the same as in this study. The transcript levels of above-mentioned house-keeping genes did not significantly differ or follow specific trends between the chemostats at various dilution rates and the retentostat (Fig. 2), thus indicating that the chosen normalization method could be safely applied to the combined transcriptome datasets. As expected from earlier chemostat studies (Regenberg *et al*., 2006; Castrillo *et al*., 2007; Brauer *et al*., 2008), a large fraction of the yeast genome (3903 genes, *P*-value<0.01, *q* < 0.0002, see Materials and methods) showed growth rate-dependent transcription in the integrated retentostat-chemostat transcriptome dataset. Genes with growth rate-dependent transcript levels were clustered in seven groups based on their growth rate-dependent transcript profiles (Fig. 3 and Supporting Information, Table S1).

### Expression of previously identified growth-rate and glucose-responsive genes at near-zero growth rates

**Fig 4:**
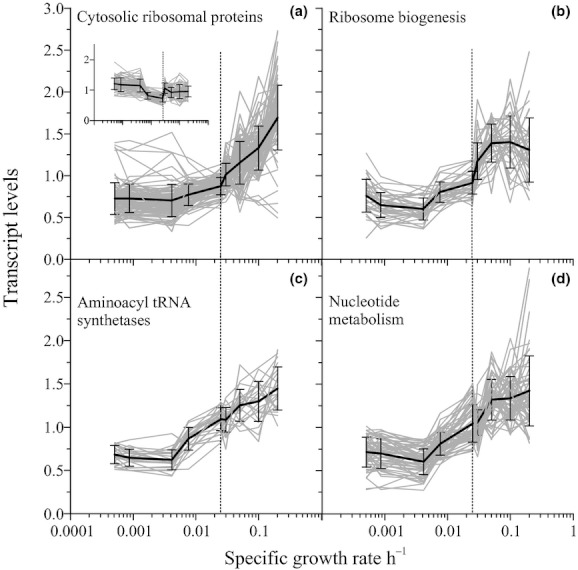
Mean-normalized expression of genes involved in protein synthesis sorted by their MIPS functional category as a function of growth rate. (a) Cytosolic ribosomal proteins. (b) RiBi (ribosomal biogenesis) cluster. (c) Aminoacyl tRNA synthetases. (d) Nucleotide metabolism. Transcript data for growth rates above 0.025 h^−1^ were obtained from Fazio *et al*. (2008) and Cipollina *et al*. (2008). Only genes with significantly changed expression (*q*-value below 0.000188, see Materials and methods) are represented. The insert in the ribosomal protein (RP) graph represents the transcript levels of genes encoding mitochondrial ribosomal proteins. Data for specific growth rates to the left of the dashed line were obtained in retentostat, whereas those to the right correspond to chemostat cultivations.

Transcript levels of many previously described growth-rate responsive genes (Regenberg *et al*., 2006; Castrillo *et al*., 2007; Fazio *et al*., 2008), also showed growth rate-dependent expression at specific growth rates below 0.025 h^−1^ (Fig. 3, Clusters 1, 2, 3, and 5). However, their transcript levels reached a constant level below a threshold specific growth rate. For example, genes encoding amino-acyl tRNA synthetases and proteins involved in nucleotide metabolism showed constant transcript levels below a specific growth rate of 0.005 h^−1^ (Fig. 4c,d). Conversely, transcript levels of genes encoding cytosolic ribosomal proteins and ribosomal-biogenesis proteins, which exhibited a strong positive correlation with specific growth rate above 0.025 h^−1^ were virtually constant below this specific growth rate (Fig. 4a,b).

**Fig 5:**
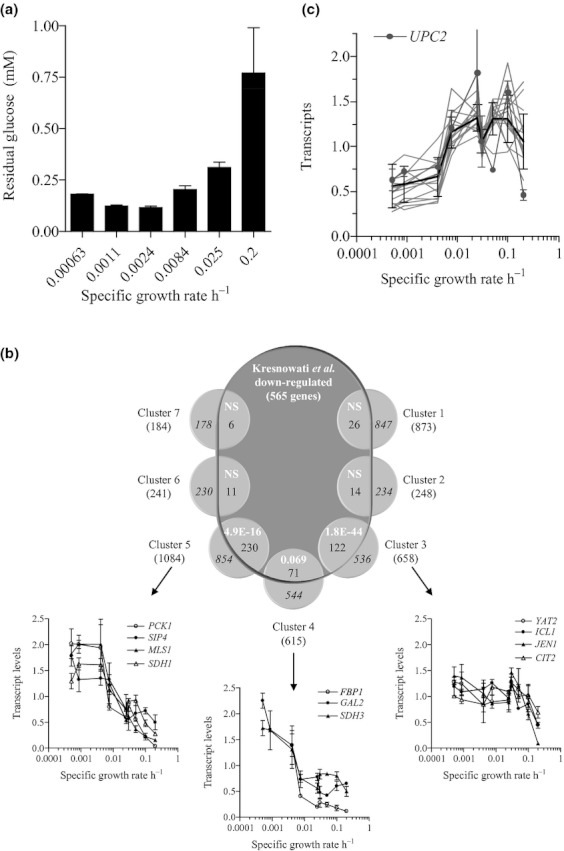
Retentostat-specific transcriptional responses. (a) Residual glucose concentration in retentostat and chemostat cultures at various growth rates. Average and mean deviation of 2–4 independent culture replicates. (b) Identification of genes responding to glucose catabolite repression using public datasets. The current dataset is compared with a set of genes shown to be repressed following glucose addition into glucose-limited yeast cultures (Kresnowati *et al*., 2006). For each cluster the total number of genes in the cluster is indicated between parentheses, the number of genes also identified in Kresnowati *et al*. is in bold and the number of genes not overlapping with Kresnowati's dataset is in italics. The significance of the enrichment in the clusters for glucose-responsive genes identified by Kresnowati *et al*. is indicated in white. NS, not significant, *P*-value cut-off 1.42E-01. For clusters 3, 4 and 5 the mean-normalized expression of well-documented targets of glucose catabolite repression is shown as a function of the specific growth rate in the combined chemostat-retentostat dataset. (c) Genes involved in ergosterol metabolism. Mean-normalized expression of 18 ERG genes expressed at various growth rates. Average expression and standard deviation is represented by the bold line.

Following Monod kinetics (Monod, 1950), in glucose-limited chemostat, the residual glucose concentration was correlated with the specific glucose consumption (*q*_glucose_) and thereby with the specific growth rate (Boender *et al*., 2009), i.e. the lower the specific growth rate, the lower the residual glucose concentration (Fig. 5a). During the course of the retentostat, the residual glucose concentration was further decreased to reach very low concentrations (Fig. 5a). As glucose repression is a key parameter in yeast transcriptional regulation, we investigated overrepresentation of a previously defined set of glucose-responsive genes (Kresnowati *et al*., 2006) in the seven gene clusters shown in Fig. 3. Glucose-repressible genes were significantly overrepresented in clusters 3, 4 and 5 (Fig. 5b). While most glucose-repressible genes (230 genes, Fig. 5b) were found in cluster 5 (including well-known glucose-repressible genes such as *PCK1*, *ADH2*, *MLS1*, *ACS1*, *SIP4*, *SDH1,2*, *SFC1* and *NDE2*), the expression profiles of genes in this cluster were surprisingly more correlated to growth rate than residual glucose concentration. The glucose-repressible genes displaying the most significant enrichment (*P*-value 1.8E-44, Fig. 5a), and the best correlation with glucose concentration were found in cluster 3. In cluster 3, the expression of 120 previously identified glucose-repressible genes was clearly de-repressed at higher specific growth rate for which the residual glucose concentration was markedly decreased from 0.78 to 0.15 mM. These genes showed a constant transcript level when the glucose concentration decreased below 0.15 mM. Typical genes in cluster 3 were *JEN1*, *CIT2*, *ICL1*, *YAT2*, *CAT2* and *CRC1*.

Surprisingly, the expression of a set of 71 glucose-repressible genes (cluster 4), of which *FBP1*, *GAL2* or *SDH3*, were not affected by changes in growth rate or in residual glucose concentration (Fig. 5b), but were specifically up-regulated near zero-growth rate.

### Retentostat and near-zero growth rate-specific responses

To explore specific transcriptional adaptations to near-zero specific growth rates, we focused on genes that were specifically up- or down-regulated at the extremely low specific growth rates that could only be studied in retentostat cultures (clusters 4, 6 and 7, Fig. 3). Unexpectedly, genes related to mitochondrial functions were strongly overrepresented among the genes in cluster 4 (Fig. 3), whose transcript levels were specifically increased at near-zero specific growth rates. This upregulation affected different mitochondrial processes: 32 of the 76 genes encoding mitochondrial ribosomal proteins (Fig. 4a, insert), respiratory chain sub-units (e.g. *ATP4, ATP7, ATP15, ATP17, COX5B*, *COX8*, *COX9*, *COX11*, *COX14*, *COX16*, *COX17*, *COX23*, *COQ5*, *COQ9*, *SOC1*, *SCO2*), protein processing (e.g. *IMP1*, *IMP2* and *SOM1*, the three subunits of the IMP complex involved in protein maturation in the intermembrane space) and mitochondrial membrane transport (*TIM17*, *TOM6*, *TPC1*, *YFH1*). This overrepresentation was unexpected as anaerobic yeast cultures cannot respire and anaerobic mitochondria only have biosynthetic, and therefore growth-related roles (Visser *et al*., 1990). Transcription of many mitochondria-related genes is under dual transcriptional control by oxygen induction and glucose repression via the Hap2/3/4/5 complex (Lascaris *et al*., 2003). Sparging with high-quality nitrogen gas and minimization of oxygen diffusion made oxygen induction extremely unlikely. The residual glucose concentration was strongly growth rate-dependent at higher specific growth rates, but was not substantially decreased for growth rates below 0.01 h^−1^ (Fig. 5a). It is, however, below this growth rate (after 13 days of retentostat) that the expression of the genes with mitochondrial functions in cluster 4 was induced, whereas the residual glucose concentration leveled off at around 0.13 mM (Fig. 5b). Furthermore, Hap4 BS were not overrepresented in the promoter regions of the genes in cluster 4. Finally, the up-regulation at near-zero growth rate of genes such as *CYC7*, *COX5B*, involved in anaerobic respiration or *ANB1*, encoding the anaerobic translation initiator factor elF5A, rule out the likelihood of an accidental increased oxygen supply in the course of the retentostat. These results suggest that up-regulation of genes encoding mitochondrial proteins at near-zero specific growth rates is not solely linked to oxygen or residual glucose concentration, but reflects a specific adaptation of yeast in extremely slow-growing (and/or ageing) cultures.

**Fig 6:**
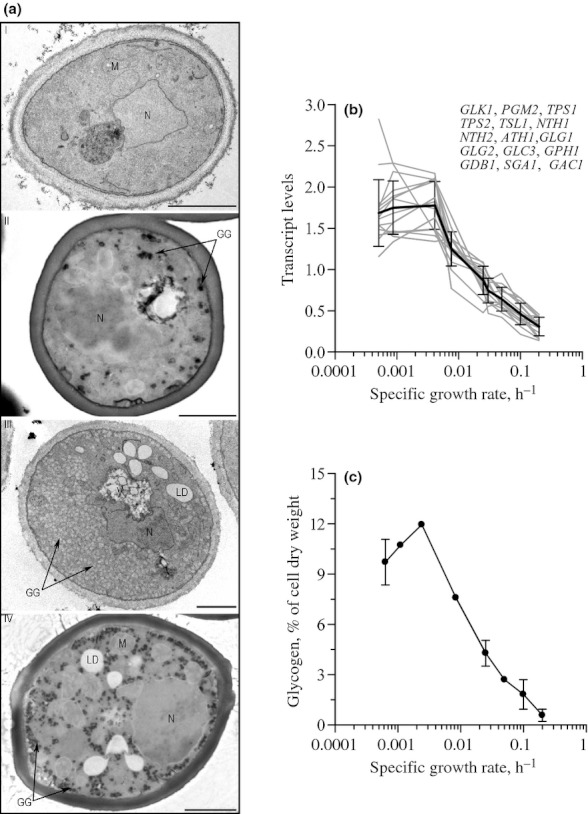
(a) Electron micrographs of *Saccharomyces cerevisiae* before starting the retentostat (pictures I and II) and after 22 days in retentostat (pictures III and IV). Glycogen was stained with phosphotungstic acid in pictures II and IV. GG, glycogen granules; LD, lipid droplets. The size bar respresents 1 μm. (b) Mean-normalized expression of genes involved in glycogen synthesis and degradation according to growth rate in the combined chemostat/retentostat dataset. (c) Intracellular glycogen contents as a function of the specific growth rate in the combined chemostat/retentostat dataset.

Among the 241 genes that showed a reduced transcript level at near-zero growth rates (cluster 6, Fig. 3), the most strongly overrepresented functional category was lipid and sterol metabolism (Table 1 and Table S2). Cluster 6 included 13 of the 19 *ERG* genes involved in ergosterol biosynthesis (Fig. 5c). Biosynthesis of sterols, which are important components of eukaryotic membranes, is strictly oxygen-dependent in *S. cerevisia*e (Servouse & Karst, 1986). Ergosterol is therefore included in media for anaerobic yeast cultivation (Andreasen & Stier, 1953; Verduyn *et al*., 1990b). As the sterol content of the medium was not adapted to the lower biosynthetic requirements of virtually non-growing cultures, it is likely that an excess of ergosterol was fed in the later stages of retentostat cultivation. The resulting increased ergosterol concentrations may have caused transcriptional down-regulation of the *ERG* genes via Upc2 or Ecm22, two sterol-responsive transcriptional activators that bind TCGTTYAG motifs (Crowley *et al*., 1996; Cohen *et al*., 2001; Vik & Rine, 2001). Consistent with this hypothesis, the *UPC2* transcript profile matched that of the *ERG* genes, and TCGTTYAG motifs were strongly overrepresented in promoter regions of genes in cluster 6 (EF = 3.8, see Materials and methods). A similar mechanism may have contributed to the down-regulation of genes involved in lipid metabolism, as the oleate ester Tween-80 is included in anaerobic yeast media to compensate for the inability of anaerobic *S. cerevisiae* cultures to synthesize unsaturated fatty acids (Andreasen & Stier, 1954). Intracellular lipid droplets observed at near-zero growth rates (Fig. 6a) may either represent ‘luxury uptake’ and storage of excess oleate from the medium or *de novo* lipid synthesis and accumulation as previously reported for quiescent cells (Matile *et al*., 1969; Rattray, 1988; Leber *et al*., 1994, see next paragraph).

**Table 1:** Enrichement for MIPS categories (primary categories indicated in capitals), KEGG pathways (indicated in italics) and TFs (indicated in bold). (see Materials and methods section. *P*-value threshold = 1E-06)

Cluster #	MIPS category, KEGG pathways and transcription factors
1	Amino acid metabolism
	Metabolism of the aspartate family
	Metabolism of methionine
	Metabolism of cysteine-aromatic amino acids
	RNA processing
	rRNA processing
	RNA modification
	rRNA modification
	PROTEIN SYNTHESIS
	Ribosome biogenesis
	Ribosomal proteins
	Translation
	Aminoacyl-tRNA-synthetases
	Nucleic acid binding
	RNA binding
	*Urea cycle and metabolism of amino acids*
	*Purine metabolism*
	*Pyrimidine metabolism*
	*Phenylalanine, tyrosine and threonine metabolism*
	*Aminoacyl-tRNA biosynthesis*
	*Ribosome*
	*RNA polymerase*
	**Sfp1**
	**Mbp1**
	**Fhl1**
	**Gcn4**
	**Rap1**
2	CELL CYCLE AND DNA PROCESSING
3	METABOLISM
	Phosphate metabolism
	Transcriptional control
	ATP binding
	CELLULAR COMMUNICATION/SIGNAL TRANSDUCTION MECHANISM
	Cellular signalling
	CELL FATE
	Cell growth/morphogenesis
	CELL TYPE DIFFERENTIATION
	Fungal/microorganismic cell type differentiation
	Fungal and other eukaryotic cell type differentiation
	Budding, cell polarity and filament formation
	*Phosphatidylinositol signalling system*
4	Mitochondrion
5	ENERGY
	CELL RESCUE, DEFENCE AND VIRULENCE
	Stress response
	UNCLASSIFIED PROTEINS
	**Msn2**
	**Aft2**
	**Skn7**
6	METABOLISM
	Lipid, fatty acid and isoprenoid metabolism
	Isoprenoid metabolism
	Tetracyclic and pentacyclic triterpenes metabolism
	*Biosynthesis of steroids*
	**Hap1**
7	*N-glycan biosynthesis*
	*Glycan structures-biosynthesis*

### Increased expression of quiescence-related genes at near-zero growth rates

Several genes involved in glycogen synthesis and degradation were strongly up-regulated at near-zero growth rates (Fig. 6b). While expression of *GSY1* and *GSY2*, which encode glycogen synthases, was not affected, *GAC1*, whose gene product activates the glycogen synthases via phosphorylation, was up-regulated at near-zero growth rate. Electron microscopy and biochemical analyses showed intracellular accumulation of glycogen at near-zero growth rates (Fig. 6a,c). Expression levels of key genes in trehalose accumulation metabolism also changed at near-zero growth rates, but intracellular trehalose levels were below detection level throughout the experiments. Very low trehalose contents have previously been reported for anaerobic chemostat cultures of *S. cerevisiae* (de Nicola *et al*., 2007; Tai *et al*., 2007; Hazelwood *et al*., 2009). Although high-level glycogen accumulation is a characteristic of quiescent *S. cerevisiae* cells, glycogen contents are also inversely correlated with specific growth rate in glucose-limited chemostat cultures grown at dilution rates above 0.025 h^−1^ (Sillje *et al*., 1997; Sillje *et al*., 1999) (Fig. 6c).

**Fig 7:**
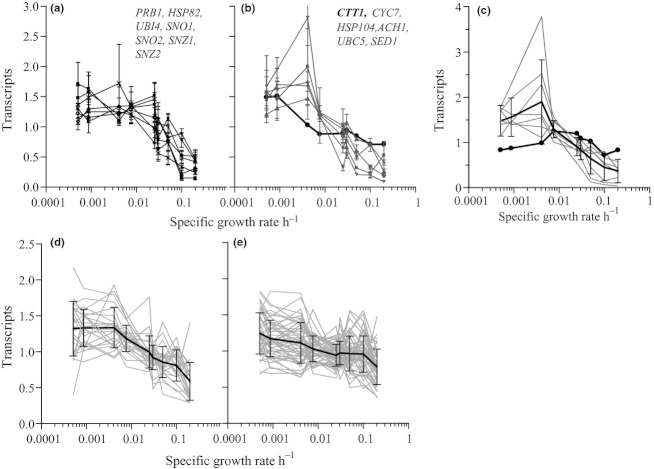
(a, b) Mean-normalized expression of quiescence-related genes (Werner-Washburne *et al*., 1993; Padilla *et al*., 1998; Shimoi *et al*., 1998). (c) Mean-normalized expression of eight G_0_-related genes. RIM15, SCH9, SSA3, HSP12, HSP26, MSN4 and GIS1 (Reinders *et al*., 1998; Pedruzzi *et al*., 2000; Swinnen *et al*., 2006) are represented as grey lines with their averaged expression as thick black line. MSN2 is represented by the black line with black dots. (d) Mean-normalized expression of the ATG genes involved in autophagy. (e) Mean-normalized expression of genes involved in ubiquitin-mediated proteolysis.

Quiescent yeast cells show increased expression of members of the *SNO* and *SNZ* genes families, mannoprotein-encoding genes such as *SED1* and the catalase encoding gene *CTT1* (Werner-Washburne *et al*., 1993; Shimoi *et al*., 1998). Several such ‘indicator genes’ for quiescence showed high expression at near-zero growth rates. However, their growth-rate-dependent expression profiles were unexpected (Fig. 7a,b). Instead of being specifically induced at (near-)zero growth rate, expression of *PRB1*, *HSP82*, *UBI4*, *SNZ1*, *SNZ2*, *SNO1* and *SNO2* was strongly inversely correlated to specific growth over a broad range of specific growth rates, and reached maximum levels below growth rates of 0.004 h^−1^ (Fig. 7a). Expression of *CYC7*, *HSP104*, *ACH1*, *UBC5* and *SED1* already levelled off below specific growth rates of 0.025 h^−1^ (Fig. 7b).

Exit of the cell cycle into G_0_ involves transcriptional up-regulation of the G_0_-specific genes *SSA3*, *HSP12* and *HSP26* (Reinders *et al*., 1998). This up-regulation is controlled by the regulators Sch9 and Rim15, whose activity is regulated by the PKA and TOR nutrient-sensing pathways (Swinnen *et al*., 2006). Sch9 and Rim15-mediated activation of G_0_ genes is mediated by binding of the TF Gis1 to the PDS box (Pedruzzi *et al*., 2000) and binding of Msn2/Msn4 to the STRE box (Martinez-Pastor *et al*., 1996). Similar to the quiescence-related genes discussed above, transcript levels of these G_0_-related genes (including the genes encoding the regulators Rim15 and Sch9) showed a negative correlation with specific growth rate over a broad range of specific growth rates and reached maximum levels at near-zero growth rates (Fig. 7c).

Genes involved in autophagy are transcriptionally upregulated in quiescent cells (Inoue & Klionsky, 2010). Autophagy is induced in response to other stress conditions and enables recycling of organelles and cellular proteins (Wang & Klionsky, 2003). Like the G_0_ genes, autophagy is under the control of the TOR pathway and nutrient sensing (Smets *et al*., 2010). Growth rate-dependent transcript profiles of autophagy-related (*ATG*) genes resembled those of other quiescence and G_0_-related genes (Fig. 7d).

### Indications for chronological ageing

In this study, retentostat cultures were studied for a period of 22 days. As, during this period, full cell retention was applied, it was of interest to explore whether some transcriptional responses observed at near-zero specific growth might in fact be related to cellular ageing. Yeast ageing can be defined in two different ways. Replicative ageing is related to the maximum number of budding events that a mother cell can go through, whereas chronological ageing is related to the maximum life span a non-dividing cell can survive (Kaeberlein *et al*., 2007). The severely constrained specific growth rate in the retentostats (only 2–3 generations in 22 days) implies a small impact on replicative ageing. However, the virtual absence of growth after the initial days of operation led to an average chronological age of cells in the retentostats of approximately 15 days at the end of the 22-day experiments.

Reactive oxygen species, which play a major role in chronological ageing in aerobic cultures (Yoshida *et al*., 2003), cannot be formed via the respiratory chain under anaerobic conditions. However, other toxic compounds may still be formed in ageing anaerobic cultures. Methylglyoxal, a non-enzymically formed by-product of glycolysis (Phillips & Thornalley, 1993; Martins *et al*., 2001; Gomes *et al*., 2005) irreversibly modifies macromolecules (DNA, RNA and proteins) via glycation (Kalapos, 1999). Expression of the methylglyoxal-inducible *GLO1* and *GRE3* genes increased during retentostat cultivation (data not shown), and d-lactate, the end-product of methylglyoxal degradation in anaerobic cultures, was produced at low levels throughout retentostat cultivation (Boender *et al*., 2009). Genes involved in both ubiquitin-mediated and ubiquitin-independent proteolysis of damaged proteins were largely non-responsive to specific growth rate in chemostat and retentostat cultures (Fig. 7e). Although a few genes involved in proteolysis (*YPS5* and *RPN4*) were specifically induced in retentostats, their expression levels remained stable throughout the final 13 days of the experiment. The transcriptome data do not therefore provide indications for extensive ageing-dependent protein damage.

**Fig 8:**
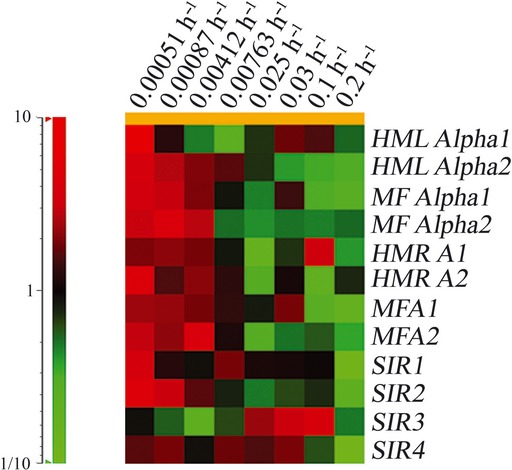
Mean-normalized expression of the SIR and HM genes as a function of specific growth rate in chemostat and retentostat cultures.

The Sir histone deacetylases fulfill two functions in yeast: (i) silencing of the mating-type loci *HML* and *HMR* and (ii) suppression of the formation of toxic rDNA circles caused by recombination at the rDNA locus (Oberdoerffer *et al*., 2008). As DNA breaks occur in ageing yeast cells, the Sir proteins, of which Sir2 is the most intensively studied, dissociate from *HM* loci and facilitate recruitment of repair factors to the break sites. This results in increased transcription of *HM* loci and expression of mating-related genes. Indeed, expression of *SIR2* and *HM* genes was specifically up-regulated after prolonged (16 days) retentostat cultivation (Fig. 8). Several additional DNA-damage-responsive genes were up-regulated at this stage, including *RNR3*, which encodes a subunit of ribonucleotide reductase (the rate-limiting enzyme in dNTP synthesis, Elledge *et al*., 1993), and three *RAD* genes (*RAD10*, *RAD24* and *RAD27*). However, most genes involved in DNA repair (RAD genes, Friedberg, 1991) were not induced during prolonged cultivation at near-zero growth rates, thereby suggesting that yeast cells did not suffer extensive ageing-dependent DNA damage.

## Discussion

Use of retentostat cultures enabled, for the first time, an analysis of the transcriptional responses of *S. cerevisiae* at extremely slow growth rates. While several chemostat-based studies have focused on specific growth rates above 0.03 h^−1^, in the present work, rates below 0.001 h^−1^ (doubling time over 600 h) were reproducibly reached and investigated. By integrating the retentostat-based data with previously obtained chemostat-based transcriptome datasets, it was therefore possible to identify growth rate-specific transcriptional patterns. The first fascinating outcome of this work was the identification of different expression patterns and of growth-rate threshold for transcription. For instance, while the expression of genes involved in specific cellular processes like translation (RP, RiBi, etc.) were not affected by growth rates below 0.025 h^−1^ (Fig. 4), related processes like amino acids synthesis, nucleotide synthesis and tRNA loading were down-regulated until growth rates of 0.005 h^−1^. These uncoupled responses of related processes suggested that the ribosomal machinery is maintained at a constant level at extremely low growth rates, whereas rates of protein synthesis are controlled by the availability of precursors for transcription and translation. While the expression of the genes involved in proteins synthesis are under the regulation of the nutrient-sensing pathways TOR and PKA, the divergent expression profiles for different protein synthesis-related processes strongly suggest that, as glucose availability dwindles, different regulatory networks fine-tune their expression. Another remarkable outcome was the specific induction of repression for growth rates below 0.008 h^−1^ of as many as 1040 genes (Fig. 3, clusters 4, 6 and 7). Unexpectedly, many genes upregulated near-zero growth rates-encoded proteins involved in mitochondrial functions. This upregulation could not be readily explained from known regulation mechanisms responding to glucose concentration or oxygen availability (Buschlen *et al*., 2003; Lascaris *et al*., 2003), as the retentostat cultures were grown under strictly glucose-limited, anaerobic conditions. Mitochondria have been involved in ageing processes (Parrella & Longo, 2008) and may be important for cell survival near zero growth-rate response to chronological ageing. In addition, even in the absence of oxygen, mitochondria fulfill various biological functions such as the synthesis of amino acids, and are suspected to be involved in more cellular processes that may be critical for yeast survival at near-zero growth rates (Visser *et al*., 1994; Reiner *et al*., 2006). The genes specifically down-regulated at near-zero growth rate were substantially enriched for functions related to sterol and lipid synthesis. This response was most probably an artefact caused by the retentostat set-up during which supplied ergosterol and fatty acids accumulate as specific growth rate decreases. Indeed, when lipid globules were stained with the dye Nile Red, the number and size of lipid globules increased in the course of the retentostat (data not shown). This technical problem can be relatively easily addressed using a decremented sterol and tween feed.

It is essential to stress that cells cultivated in retentostat are not starved as they receive a continuous, albeit low, carbon and energy supply. Although attained artificially in retentostat, these cultivation conditions are very relevant as they mimick natural environments frequently encountered by microbes, such as the slow release of assimilable carbon source from rotting material or by chemical conversion of complex raw material. While a wealth of data describe the physiology of yeast cultures starved for carbon, i.e. quiescent cells, very little is known about the physiological status of cells at extremely slow growth rates. One of the most puzzling aspects of these very slowly dividing cells concerns their cell cycle phase. In starved cultures that have reached stationary phase, cells become quiescent and exit the cell cycle to rest in G_0_ phase. Cells in retentostat display features associated with quiescent cells, i.e. accumulation of glycogen and lipid, down-regulation of protein synthesis, upregulation of genes implicated in autophagy and G_0_, upregulation of *SNO* and *SNZ* gene families. However, although quiescent cells have been shown to retain some residual metabolic activity (Gray *et al*., 2004), cells in retentostat maintain substantial catabolic and anabolic activity as revealed, on one hand, by their substantial product formation rates (ethanol *c.* 1 mmol g^−1^ h^−1^, Boender *et al*., 2009) and, on the other hand, by their remarkable survival after 22 days of retentostat (Fig. 1). It has been shown that slowly growing cells spend a substantial part of their cell cycle in G_1_ phase (Sillje *et al*., 1997). It is therefore reasonable to consider that cells in retentostat survive in extended G_1_ phase. However, although in line with the substantial metabolic activity observed, this scenario does not agree with the strong induction of G_0_ hallmarks at near-zero growth rates. The presence of a heterogeneous population could reconcile both scenarios. Indeed, when a fraction of a growing culture would exit the replicative cell cycle at G_0_, part of the population would become quiescent while enabling the remainder of the culture to grow faster than the average growth rate of the entire population. This type of heterogeneity would differ from the heterogeneity reported in stationary-phase cultures, in which a fraction of the culture displays quiescence features, whereas the remaining cells appear to be apoptotic (Allen *et al*., 2006). When observed by phase contrast microscopy, carbon starved cultures are characterized by the appearance of dark cells that seem to display apoptotic features (Allen *et al*., 2006). In the course of the retentostat, the large majority of cells (above 99%) remained phase-dark light. We have further explored the possibility of culture heterogeneity by fusing promoters of the G_0_-related genes *SSA3* and *HSP26* with a green-fluorescent-protein (GFP)-encoding gene. However, the oxygen requirement for fluorescence of GFP precluded its effective use in the anaerobic cultures used in this study (data not shown). Aerobic retentostat experiments and/or use of a recently developed fluorescent reporter system that functions under anaerobic conditions (Drepper *et al*., 2007) may help to resolve this issue.

Unexpectedly, while genes that were previously implicated in quiescence and G_0_ showed maximum transcript levels at near-zero specific growth rates, their transcriptional upregulation was not exclusive to virtually non-growing retentostat cultures. Instead, their transcripts increased over a broad range of decreasing specific growth rates, with increased transcript levels already evident at specific growth rates as high as 0.05 h^−1^ (corresponding to a doubling time of 14 h). Up-regulation of genes involved in autophagy, down-regulation of protein synthesis and increased heat-shock tolerance have been shown to be correlated with specific growth rate in several previous studies on aerobic chemostat cultures (Elliott & Futcher, 1993; Regenberg *et al*., 2006; Brauer *et al*., 2008; Lu *et al*., 2009; Klosinska *et al*., 2011). One possible explanation for these observations is related to culture heterogeneity. Indeed, the increased expression of G_0_-related genes may result from an increasing proportion of the population exiting the cell cycle as the growth rate decreases. However, the expression of quiescence-related genes is already responding to growth rate below 0.1 h^−1^ in exponentially growing chemostat cultures (Fig. 1a,b,c), which would suggest that a substantial fraction of exponentially growing cultures have exited to G_0_. Although our results are limited to transcription, Lu *et al*. (2009) concluded that heat-shock resistance, so far an attribute of quiescent cells, was in reality growth-rate dependent. Cell heterogeneity in chemostat cultures has received little attention (Sillje *et al*., 1997; Sillje *et al*., 1999) and the extent and nature of population heterogeneity still have to be uncovered. If the expression of quiescence-related functions in slow-growing cultures does not reflect culture heterogeneity, this would indicate that decreasing growth rates result in a gradual transition rather than a sharp transition into quiescence. This would then imply a clear separation of physiological characteristics associated with quiescence (e.g. increased robustness, accumulation of storage materials) from the exit of the replicative cell cycle. This possibility was already raised by Elliott & Futcher (1993) who, based on studies on heat-shock resistance in batch cultures grown on various carbon sources, proposed that ‘the heat-shock resistance of stationary-phase cells may simply be the extreme end of a continuum’. In such a situation, exit to G_0_ may be the ultimate phase of a continuum of a gradual adaptation to lower specific growth rates. Such a gradual adaptation to slow growth would be entirely compatible with the involvement of general sensing mechanisms that, via the PKA and TOR regulatory pathways, regulates many cellular features implicated in quiescence in response to nutrient availability (Pedruzzi *et al*., 2003; Roosen *et al*., 2005; Swinnen *et al*., 2006). In line with our and Elliot and Futcher's observations, in a recent work combining chemostat cultivations at various growth rates above 0.05 h^−1^, Klosinska *et al*. (2011) suggested that the transcriptional re-programming observed during starvation ‘is in large part a direct extrapolation of the changes that occur during slow growth’. Until a possible role of culture heterogeneity has been resolved, increased expression of transcripts that were previously implicated in G_0_ (e.g. *SSA3, HSP12* and *HSP26*, Reinders *et al*., 1998) cannot be used as a reliable indicator for an actual exit of the replicative cell cycle. The present study stresses the need for dissecting the robust physiological state of slow-growing cultures from the actual exit of the replicative cell cycle into G_0_.

Cells in retentostat do not, or hardly, divide, but remain metabolic active and thereby strongly resemble post-mitotic cells found in metazoan (e.g. neurons, muscle cells and many glandular cells). *Saccharomyces cerevisiae*, because of its similarity to higher eukaryotes and its ease to cultivate and genetically manipulate, has extensively been used as model organism for ageing studies. Chronological ageing, i.e. the calendar time that an undividing cell can survive in stationary phase, is typically investigated in cells that are starved after exhaustion of the carbon source. As previously argued, these conditions hardly emulate ageing in metazoan cells (Gershon & Gershon, 2000a; Laun *et al*., 2006). Indeed, under these circumstances, responses to complete absence of external carbon source and the resulting fast cellular deterioration are investigated rather than life span. Longevity of cells in retentostat is strongly extended when compared with that of cells in stationary phase in similar media. Indeed, over 50% of the cells were able to divide after 22 days of retentostat, whereas stationary-phase cells maintained in synthetic medium typically loose over 80% of their viability within 10 days (Fig. 1, Fabrizio *et al*., 2003; Parrella & Longo, 2008). This longevity is also supported by the absence in retentostat cultures of a significant population of apoptotic cells, as indicated by the absence of ‘phase-dark’ cells (Allen *et al*., 2006) in phase-contrast microscopy. The comparative lack of deterioration in the retentostat cultures was also evident from the weak transcriptional upregulation of systems involved in DNA and protein repair. This increased longevity can be explained by the continuous supply of carbon, and energy source enabled the continuation of repair and maintenance processes, even in a virtually non-growing culture. The continuous, but highly restricted nutrient supply to retentostats resembles post-mitotic cells in human tissues much more closely than starvation in batch cultures. Finally, the absence of oxygen in the anaerobic retentostats is likely to have prevented oxidative damage, which is a major factor in ageing in aerobic cultures (Osiewacz & Scheckhuber, 2006). As the oxygen supply in bioreactors can be easily tuned and monitored, retentostat culture is also a powerful tool to investigate oxygen-related damage and their role in ageing.

## Statement

Re-use of this article is permitted in accordance with the Terms and Conditions set out at http://wileyonlinelibrary.com/onlineopen#OnlineOpen_Terms

## Supporting Information

**Table S1.** Combined chemostat and retentostat datasets

**Table S2.** Enrichment analysis for functional categories and transcription factors binding.
